# Supplementary material for: Interoperable Nanoparticle Sensor Capable of Strain and Vibration Measurement for Rotor Blade Monitoring
Source: Sensors (Basel). 2021 May 24;21(11):3648. doi: 10.3390/s21113648 (PMC8197258; doi:10.3390/s21113648)
Supplement: Supplementary file 1 [file sensors-21-03648-s001.zip › sensors-1121940-supplementary.pdf]

Interoperable Nanoparticle Sensor capable of Strain and Vibration Measurement for Rotor Blade Monitoring

Soo-Hong Min[1], Ying-Jun Quan[2], Suyoung Park[1], Gil-Yong Lee[3], Sung-Hoon Ahn[*,1,2]

1 Department of Mechanical Engineering, Seoul National University, Seoul 08826, Republic of Korea

2 Institute of Advanced Machines and Design, Seoul National University, Seoul 08826, Republic of Korea

3 Department of Mechanical Engineering, Kumoh National Institute of Technology, Gumi, Gyeongbuk 39177, Republic of Korea

*Corresponding author: ahnsh@snu.ac.kr

S1. Previous researches of sensor according to measurable range and gauge factor.

S2. Previous researches of multivariate measurable sensor.

S3. Material-Substrate Compatibility of AFN printing process.

S4. Mechanical Crack Generation.

S5. Process reproducibility of AFN printing process.

S6. Life-cycle evaluation test of AFN printed sensor.

S7. Environmental test of AFN printed sensor.

S8. The statistical results of printed sensor according to induced frequency.

S9. Resistance measurement method in rotating environment.

S1. Previous researches of sensor according to measurable range and gauge factor.

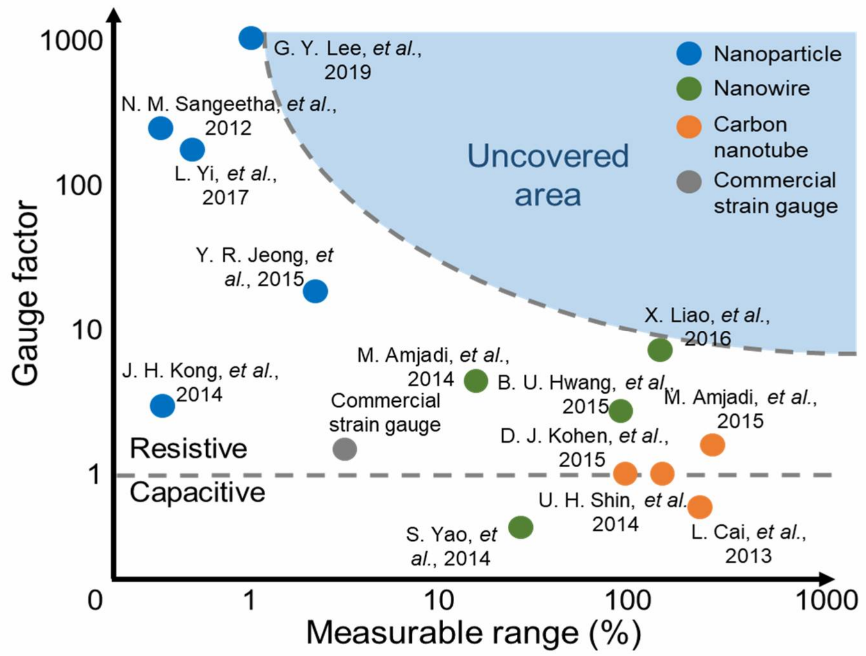

Figure S1. Previous researches of sensor according to measurable range and gauge factor [S1-S12].

S2. Previous researches of multivariate measurable sensor.

Table S1. Previous researches of multivariate measurable sensor [S7, S9, S10, S13, S14].

Previous studies	Gauge factor	Measurable range (%)	Sensing type
C. S. Boland, et al., 2014	3.5	80	Strain + Vibration
X. Liao, et al., 2016	9.65	150	Strain + Temperature
U. H., Shin, et al., 2014	1	150	Strain + Pressure
S. Yao and Y. Zhu, 2014	0.7	50	Strain + Pressure
W. Fan, et al., 2016	-	100	Strain + Temperature, UV, Electrocardiogram

S3. Material-Substrate Compatibility of AFN printed process.

Printing quality of AFN printing process was evaluated with four different level where X, Δ, □, and O denote not deposited, scattered, deposited, and patterned, respectively as shown in table S2. This study focused on AgNPs which was well deposited onto relative hard substrates such as commercial PET, Kapton polyimied film, and commercial silicon wafer treated by hydrogen fluoride (HF).

In terms of carbon fibre reinforced plastic (CFRP) and glass fibre reinforced plastic (GFRP), since FRP usually has directional properties, AgNPs was printed onto saddle between each fibre structure. However, major obstacle of using CFRP or GFRP as a substrate of sensor is their electrical conductivity which disturbs measuring electrical resistance of printed conductive nanomaterial pattern separately.

However, soft substrates such as polydimethylsiloxane (PDMS) showed completely different results. Specifically, Sylgard 184 (Dow Corning, Inc., Midland, MI) and EcoFlex 0030 (Smooth-on, Inc., Macungie, PA) were used which are types of PDMS with low elastic modulus of 1.84 MPa and 0.07 MPa, respectively. AgNP bounced back when it collided with soft substrates and not deposited.

Table S2. Material-substrate compatibility of AFN printing process (Results added from Kim, C. S., et al., 2013)

	CFRP	GFRP	PI	PDMS	PCB	PE	PP	PMMA
TiO_2	X					Δ	Δ	□
Al_2O_3								
BTO								
CNT	Δ	Δ	X	O				
Sn					O			O
Ni					Δ	Δ		Δ
Al							□	
Cu	X				X			
AlN								
Ag	O	O	O	X				
Ag/CNT	Δ	Δ	X	O				

	PET	AlSl	Cu	Al	Ni	NiTi	Si	SiO_2
TiO_2	□	□	□	□	□		□	□
Al_2O_3		□	□	□			□	□
BTO							O	
CNT	X						X	
Sn	O		O	O	O	O	O	O
Ni	Δ	Δ					O	
Al			Δ					
Cu								
AlN			□	□				
Ag	O						O	
Ag/CNT	X						X	

S4. The statistical results of printed sensor according to induced frequency.

According to previous research, it was observed that the generation of mechanical cracks along its length by gradually increase the applied strain to the sensor [S1]. In experiments, these cracks can be clearly recognized under the applied strain exceeds 1.5 to 2 %. Figure S2 presents magnified images of mechanical cracks captured by SEM after 4% of strain is applied to the sensor. The cracks are created uniformly through the length direction, and they are separated by around 76 μm in average.

The mechanical cracks are basically generated if tensile force applied to the sensor exceeds the adhesive force between nanoparticles. The magnitude of adhesive force is usually governed by the porosity of printed pattern, the pattern with lower porosity shows lower adhesive force and cracks easily. It was also demonstrated experimentally in previous research [S1].

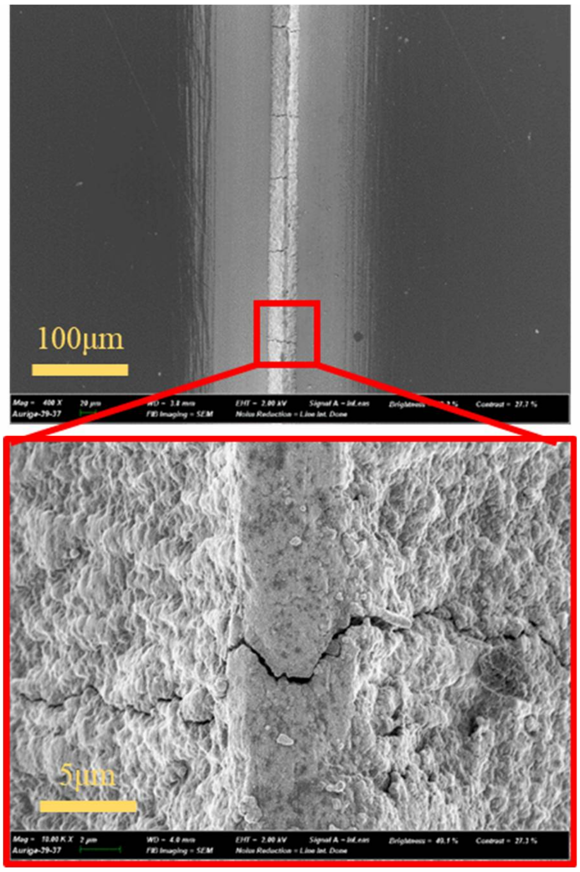

Figure S2. SEM image of mechanical crack in developed sensor.

S5. Process reproducibility of AFN printing process.

To evaluate the process reproducibility of AFN printing process, power spectrum was measured according to vibration frequency as shown in figure S3. Ten sensors were fabricated by AFN printing process with identical process parameters and attached to vibration shaker. Then, average and standard deviation of amplitude spectrum were calculated from 100 Hz to 1000 Hz in units of 100 Hz. Experimental results exhibited that deviation of amplitude spectrum was not exceeded 10% at all tested frequencies.

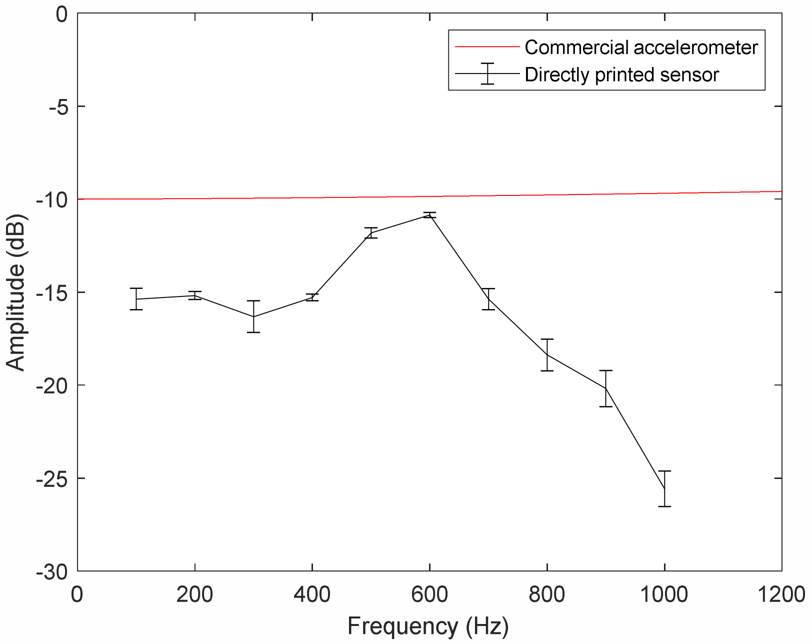

Figure S3. The average and standard deviation value of vibration amplitude by frequency of 10 different sensors.

S6. Life-cycle evaluation test of AFN printed sensor.

First, life cycle evaluation test for strain sensing to evaluate the mechanical stability. Figure S4 shows life cycle evaluation test results for printed sensor. Life cycle evaluation test was conducted by 1,000 times of loading and unloading tests. After 10 – 20 cycles to stabilised, printed sensor remained stable and the variance in peak resistance was under 5%. The life cycle evaluation test for vibration sensing was also conducted. Figures S5 and S6 show the relative resistance change and its RMS value of sensor signal during seven hours. According to table S3, the RMS value during life cycle evaluation test remain with small deviation value which demonstrates the robustness of printed sensor.

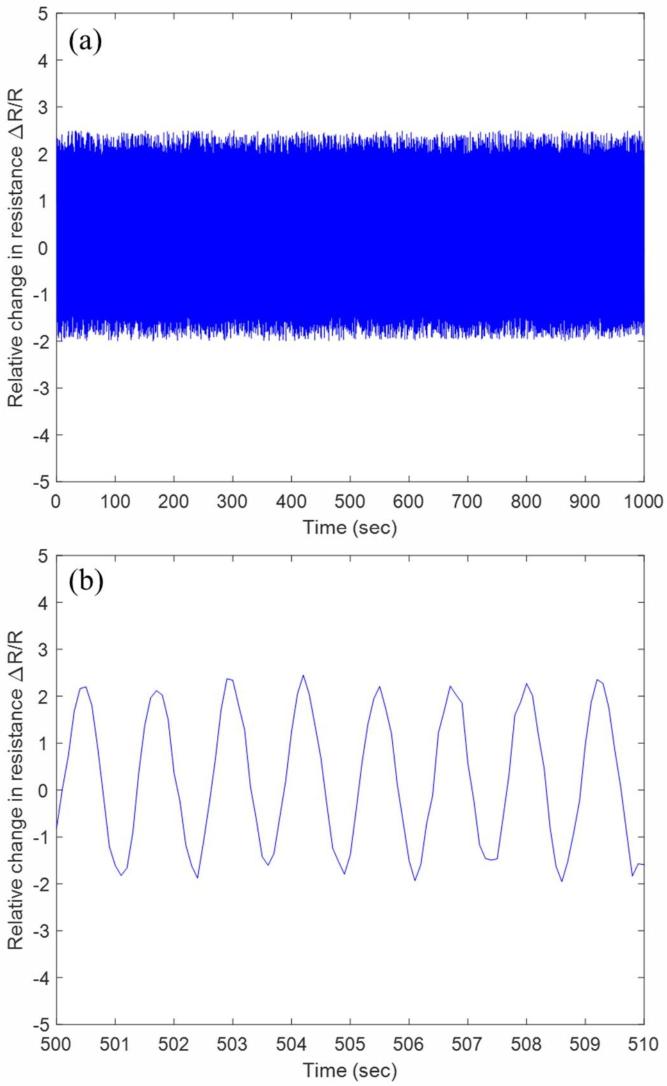

Figure S4. (a) The evaluation results of life-cycle test for strain sensing and (b) its magnified view [S15]

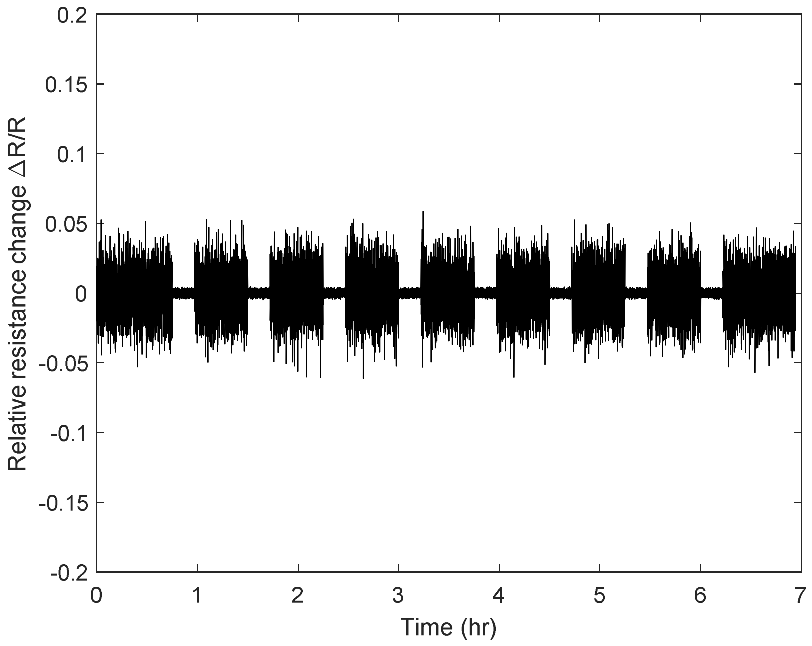

Figure S5. The evaluation results of life cycle evaluation test of vibration sensing.

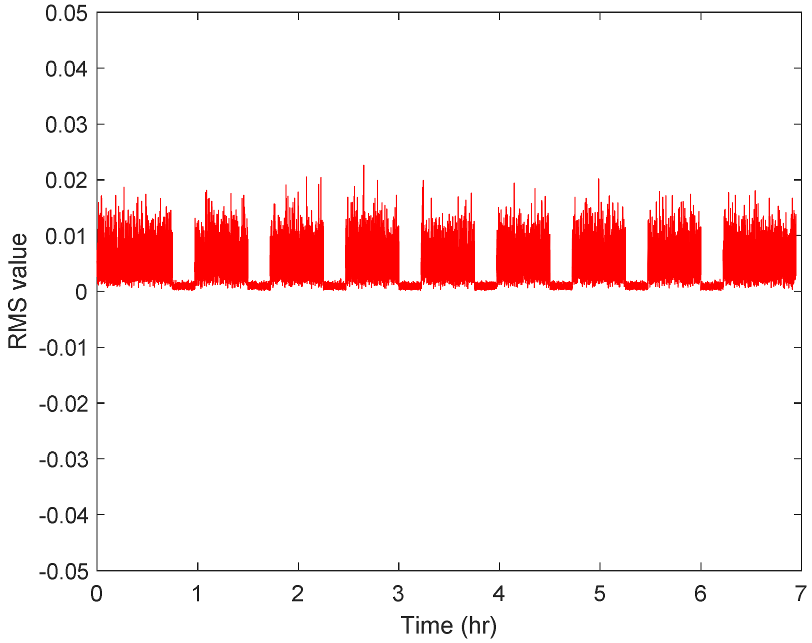

Figure S6. The RMS value of life cycle evaluation test of vibration sensing.

Table S3. The statistical analysis of RMS value of life cycle evaluation test of vibration sensing.

Average	Deviation	Maximum	Minimum
0.0078	0.0024	0.0208	0.0050

S7. Environmental test of AFN printed sensor.

To analyse the change of resistance of printed sensor according to temperature and humidity, respectively, the temperature-humidity chamber was utilised with data acquisition device. The relative resistance change decrease until around 50°C and increase after as shown in figure S7. The increase of relative resistance after 50°C was well followed the original properties of AgNPs in terms of thermal coefficient in both heating and cooling process. Hence, the reversibility of AFN printed sensor until sintering temperature was well verified. Relative resistance also decrease by relative humidity of chamber as shown in figure S8. It is assumed that the decrease was due to the increase of potential barrier between nanomaterials by water molecules in nanogaps which behaved as a huddle to the motion of electrons.

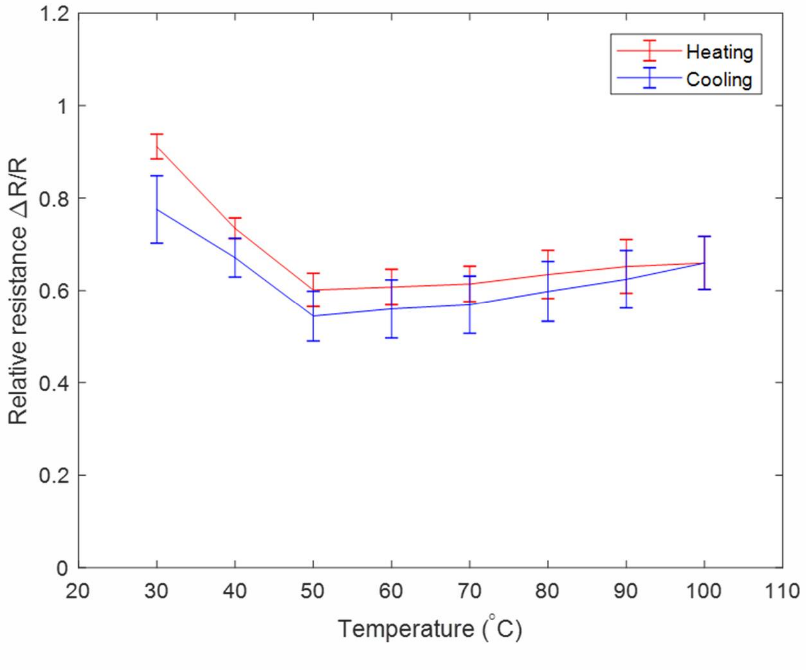

Figure S7. The relative resistance of printed sensor according to temperature.

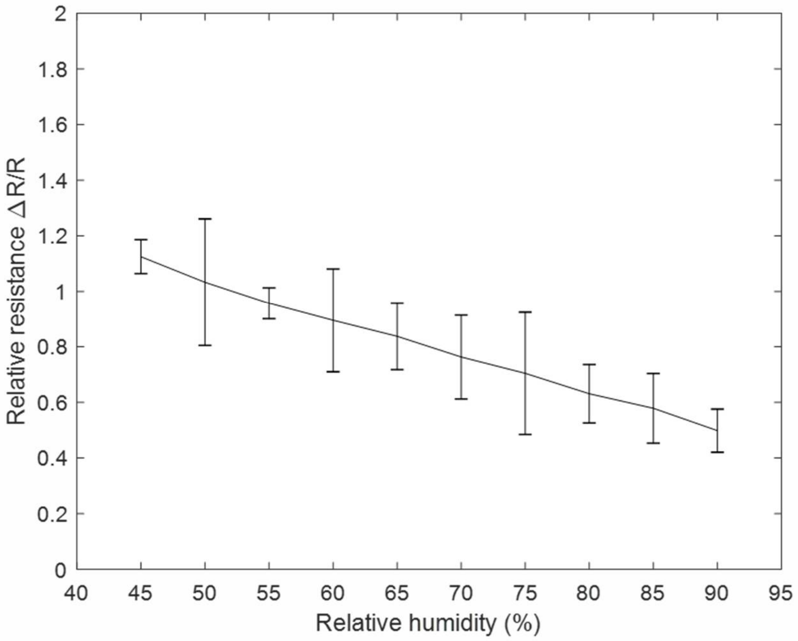

Figure S8. The relative resistance of printed sensor according to humidity.

S8. The statistical results of printed sensor according to induced frequency.

Table S4. The statistical results according to induced frequency with 200 Hz.

Source	Degree of freedom	Sum of squares
$f_0 = 0$	1	3.78
$f_1 = 100$	2	9.19
$f_2 = 200$	2	50.72
$f_3 = 300$	2	9.74
$f_4 = 400$	2	4.75
$f_5 = 500$	2	4.69
$f_6 = 600$	2	4.57
	13	87.44

Table S5. The statistical results according to induced frequency with 400 Hz.

Source	Degree of freedom	Sum of squares
$f_0 = 0$	1	1.59
$f_1 = 100$	2	5.6
$f_2 = 200$	2	5.02
$f_3 = 300$	2	3.96
$f_4 = 400$	2	48.04
$f_5 = 500$	2	12.32
$f_6 = 600$	2	1.83
	13	78.36

Table S6. The statistical results according to induced frequency with 600 Hz.

Source	Degree of freedom	Sum of squares
$f_0 = 0$	1	7.65
$f_1 = 100$	2	1.19
$f_2 = 200$	2	5.73
$f_3 = 300$	2	5.4
$f_4 = 400$	2	8.78
$f_5 = 500$	2	8.54
$f_6 = 600$	2	48.63
	13	85.92

S9. Resistance measurement method in rotating environment.

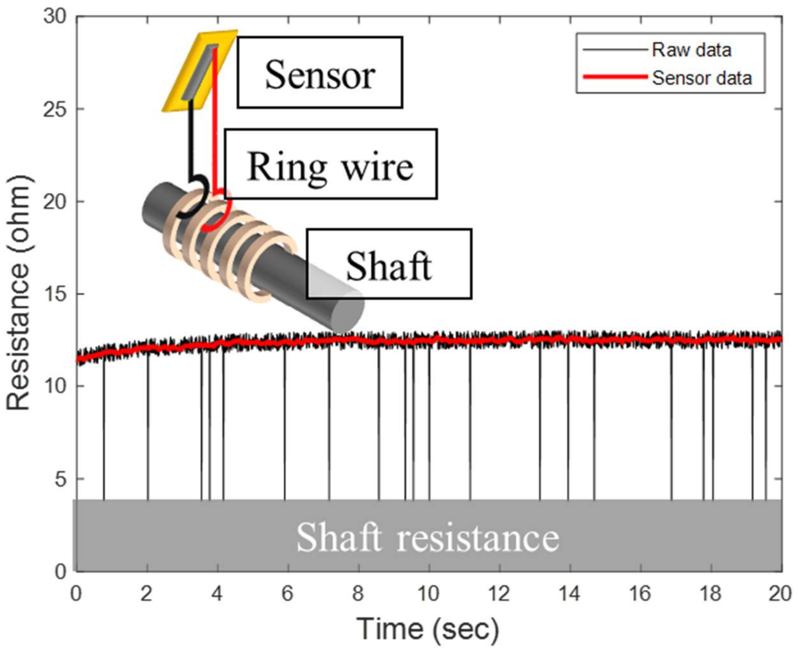

Figure S9. Schematic diagram of the resistance measurement method in rotating environment.

Figure S9 shows the schematic diagram of the resistance measurement method in rotating environment. Since the electrical wires to measure the resistance usually twisted when measure a rotating object, we used the wire with a loop shape and hooked to the ring wire connected to the shaft. By measure the resistance of the shaft and subtracting the original shaft resistance, we could estimate the resistance of the sensor itself. The resistance which was not properly measured during rotation, was inferred through linear interpolation by comparing the values before and after.

REFERENCES

[S1] Lee, G. Y., et al., Highly Sensitive Solvent-free Silver Nanoparticle Strain Sensors with Tunable Sensitivity Created using an Aerodynamically Focused Nanoparticle (AFN) Printer, ACS applied materials and interfaces, 2019, 11(29): 26421-26432.

[S2] Sangeetha, N. M., et al., Nanoparticle-Based Strain Gauges Fabricated by Conductive Self Assembly: Strain Sensitivity and Hysteresis with Respect to Nanoparticle Sizes, The Journal of Physical Chemistry C, 2013, 117: 1935-1940.

[S3] Yi, L., et al., Nanoparticle monolayer-based flexible strain gauge with ultrafast dynamic response for acoustic vibration detection, Nano Research, 2015, 8(9): 2978-2987.

[S4] Jeong, Y. R., et al., Highly stretchable and sensitive strain sensors using fragmentized graphene foam. Advanced Functional Materials, 2015, 25(27): 4228-4236.

[S5] Kong, J. H., et al., Simple and rapid micropatterning of conductive carbon composites and its application to elastic strain sensors, Carbon, 2014, 77:199-207.

[S6] Amjadi, M., et al., Highly Stretchable and Sensitive Strain Sensor Based on Silver Nanowire – Elastomer Nanocomposite, ACS Nano, 2014, 8(5): 5154-5163.

[S7] Yao, S., and Zhu, Y., Wearable multifunctional sensors using printed stretchable conductors made of silver nanowires, Nanoscale, 2014, 6(4): 2345-2352.

[S8] Hwang, B. U., et al., Transparent stretchable self-powered patchable sensor platform with ultrasensitive recognition of human activities, ACS Nano, 2015, 9(9): 8801-8810.

[S9] Shin, U. H., et al., Highly stretchable conductors and piezocapacitive strain gauges based on simple contact-transfer patterning of carbon nanotube forests, Carbon, 2014, 80: 396-404.

[S10] Liao, X., et al., A Highly stretchable ZnO@ Fiber-Based Multifunctional Nanosensor for Strain/Temperature/UV Detection, Advanced Functional Materials, 2016, 26(18): 3074-3081.

[S11] Cai, L., et al., Super-stretchable, Transparent Carbon Nanotube-Based Capacitive Strain

Sensors for Human Motion Detection, Scientific Reports, 2013, 3: 3048.

[S12] Amjadi, M., Yoon, Y. J., and Park, I., Ultra-stretchable and skin-mountable strain sensors using carbon nanotubes-Ecoflex nanocomposite, Nanotechnology, 2015, 26: 375501

[S13] Boland, C. S., et al., Sensitive, high-strain, high-rate bodily motion sensors based on graphene-rubber composties, ACS Nano, 2014, 8(9): 8819-8830.

[S14] Fan, W., Bu, W., and Shi, J., On the Latest Three-Stage Development of Nanomedicines based on Upconversion Nanoparticles, Advanced Materials, 2016, 28(21): 3987-4011.

[S15] Min, S.H., et al., Direct printing of highly sensitive, stretchable, and durable strain sensor based on silver nanoparticles/multi-walled carbon nanotubes composites. Composites Part B: Engineering, 2019, 161, 395-401.

[S16] Min, S.H., et al.,
